# Impact of Helminth Infection on Metabolic and Immune Homeostasis in Non-diabetic Obesity

**DOI:** 10.3389/fimmu.2020.02195

**Published:** 2020-09-16

**Authors:** Anuradha Rajamanickam, Saravanan Munisankar, Kannan Thiruvengadam, Pradeep A. Menon, Chandrakumar Dolla, Thomas B. Nutman, Subash Babu

**Affiliations:** ^1^National Institute of Health-National Institute for Research in Tuberculosis (NIRT)-International Center for Excellence in Research, Chennai, India; ^2^Department of Epidemiology, National Institute for Research in Tuberculosis, Chennai, India; ^3^Laboratory of Parasitic Diseases, National Institute of Allergy and Infectious Diseases, National Institutes of Health, Bethesda, MD, United States; ^4^Frederick National Laboratory for Cancer Research Sponsored by the National Cancer Institute, Frederick, MD, United States

**Keywords:** strongyloides stercoralis infection, obesity, adipocytokines, pancreatic hormones, cytokines

## Abstract

Several epidemiological and immunological studies indicate a reciprocal association between obesity/metabolic syndrome and helminth infections. Numerous studies demonstrated that obesity is concomitant with chronic low-grade inflammation, which is marked by vital changes in cellular composition and function of adipose tissue. However, the effect of helminth infection on the homeostatic milieu in obesity is not well-understood. To determine the relationship between *Strongyloides stercoralis (Ss)* infection and obesity, we examined an array of parameters linked with obesity both before and at 6 months following anthelmintic treatment. To this end, we measured serum levels of pancreatic hormones, incretins, adipokines and Type-1, Type-2, Type-17, and other proinflammatory cytokines in those with non-diabetic obesity with (INF) or without *Ss* infection (UN). In INF individuals, we evaluated the levels of these parameters at 6 months following anthelmintic treatment. INF individuals revealed significantly lower levels of insulin, glucagon, C-peptide, and GLP-1 and significantly elevated levels of GIP compared to UN individuals. INF individuals also showed significantly lower levels of Type-1, Type-17 and other pro-inflammatory cytokines and significantly increased levels of Type-2 and regulatory cytokines in comparison to UN individuals. Most of these changes were significantly reversed following anthelmintic treatment. *Ss* infection is associated with a significant alteration of pancreatic hormones, incretins, adipokines, and cytokines in obese individuals and its partial reversal following anthelmintic treatment. Our data offer a possible biological mechanism for the protective effect of *Ss* infection on obesity.

## Introduction

Obesity and metabolic disorders are major public health problems because of their high prevalence worldwide. In 2016, ~1.9 billion adults were overweight and of these 650 million were obese (1). In India, more than 135 million people are suffering from obesity (2). Obesity is described as unequal body weight for height with an extra deposit of adipose tissue along with low-grade chronic and systemic inflammation (3). Obesity induced inflammation can lead to the development of type 2 diabetes, cardiovascular disease, liver disease, certain type of cancers and other pathological conditions (4, 5).

Obesity is linked with an abnormal expansion in adipose tissue mass and adiposity, as well as poorly regulated levels of adipokines and dysregulation of Type-1 and Type-2 cytokines (6). Adipose tissue aids in energy storage and secretes adipokines—adiponectin, leptin, tumor necrosis factor-α (TNF-α), resistin, and plasminogen-activator type 1 (PAI-1) (7). Obese adipose tissue primarily releases proinflammatory cytokines such as TNF-α, IL-6, leptin, visfatin, resistin, and plasminogen activator inhibitor-1. Obesity activates an immune response which incorporates a systemic elevation of inflammatory cytokines, the recruitment of immune cells to inflamed tissues, activation of leukocytes, and the generation of repair tissue responses (8).

Helminth infections affect approximately one-quarter of the world's population and are widespread in lower to middle-income countries (9). The occurrence of obesity is commonly predominant in urbanized countries where most helminth infections have been eliminated (10, 11). Recent data in both animal and human studies showed a reciprocal association between helminth infection and metabolic disorders, type-2 diabetes, insulin resistance and obesity (12–16), suggesting that helminths may have role in the prevention or delay of these diseases. In previous studies, we have shown that glycemic, hormonal, and cytokine factors in T2DM individuals are modulated by *Strongyloides stercoralis* infection and these changes are partially reversed following anthelmintic therapy (17). Additionally, we have also shown that T2DM with *Ss* infected individuals exhibited significantly lower systemic levels of cytokines and chemokines and dampens the pro-inflammatory milieu, an effect which is then reversed upon anthelmintic treatment (18). However, the mechanisms of how helminth infections mediate protection against non-diabetic obesity are unknown.

Therefore, in the current study, we wanted to examine the association among *Ss* infection and non-diabetic obesity and assessed the influence of *Ss* infection on factors essential in adipose tissue homeostasis. To this end, we estimated systemic levels of pancreatic hormones (insulin, glucagon and C-peptide), incretins (Ghrelin, GIP, GLP-1), adipokines (adiponectin, adipsin, resistin, leptin, visfatin and PAI-1) and a variety of cytokines, including Type-1 (IFN-γ, IL-2, and TNF-α), Type-17 (IL-17A and IL-22), Type-2/regulatory cytokines (IL-4, IL-5, IL-13, and IL-10) and other pro-inflammatory cytokines (IL-1α, IL-1β, IL-6, IL-12, and GM-CSF) in those with obesity with or without concomitant *Ss* infection. We also examined the consequence of anthelmintic therapy on the above-mentioned factors in *Ss*-infected individuals.

## Methods

### Ethics Statement

The study protocol was (12-I-073) permitted by Institutional Review Boards of the National Institute of Allergy and Infectious Diseases (USA) and the National Institute for Research in Tuberculosis (India) (approval no. NCT00375583 and NCT00001230). All individuals were screened as part of a natural history study protocol, and informed written consent was acquired from all individuals.

### Study Population

We enrolled 115 study participants comprising of 58 clinically asymptomatic *Ss*-infected study participants with obesity (hereafter INF), and 57 study participants with obesity and negative for *Ss* infection (hereafter UN) in Kanchipuram District, Tamil Nadu, South India (Table 1). None had previous anthelmintic treatment, a history of helminth infections or of HIV. Individuals with diabetes or prediabetes (HbA1c above 5.7%) were excluded. These individuals were different from the individuals recruited in our previous studies (17, 18).

**Table 1:** Demographic and biochemical parameters.

	**Ss+**	**Ss-**	
	***n* = 58**	***n* = 57**	***p*-values**
M/F	30/28	28/29	0.8528
Age	36 (20–64)	39 (24–64)	0.1975
BMI (kg/m^2^)	31 (30–36)	34 (30–40)	0.8073
RBG (mg/dl)	96 (70–168)	99 (69–159)	0.4700
HbA1c (%)	5.2 (5.1–5.5)	5.3 (5.2–5.6)	0.3774
Urea (mg/dl)	19.8 (8–40)	22.1 (13–42)	0.2972
Creatinine (mg/dl)	0.72 (0.3–1.2)	0.75 (0.4–1.3)	0.4448
ALT (U/L)	19.5 (10–116)	20.1 (5–87)	0.9766
AST (U/L)	22.4 (14–58)	23.8 (14–130)	0.4609

***INF** refers to individuals with Ss-infection with obesity, **UN** refers to individuals with obesity without Ss infection. For age and gender p-value calculated using chi square (and Fisher's exact) test. Other parameter's p-values calculated using Mann-Whitney U-test*.

### Measurement of Anthropometric and Biochemical Parameters

Anthropometric measurements, including height, weight, waist circumference and biochemical parameters, including plasma glucose, lipid profiles and HbA1c were obtained using standardized techniques as detailed elsewhere (19).

### Parasitological Examination and Anthelmintic Treatment

The recombinant NIE antigen-ELISA detects IgG antibodies and was performed for the identification of *Ss* infection, as described previously (20, 21). Single stool specimens were collected at baseline from all screened individuals before anthelmintic therapy. Other intestinal helminth infections were identified by Stool microscopy and expelled from the study. Circulating filarial antigen tests were used for the diagnosis of filarial infection and those who were positive were excluded from the study. A single dose of ivermectin (12 mg) and albendazole (400 mg) were given to the INF study participants. Following 6 months of anthelmintic treatment, stool and blood samples were collected from the treated individuals to check the IgG antibody levels and to identify the presence of helminth infection. All INF individuals exhibited reductions in IgG titers at this time point and no helminths were detected by microscopy.

### Determination of Obesity

BMI more than or equal to 30 kg/m^2^ is described as obesity based on The American Association of Clinical Endocrinologists/American College of Endocrinology algorithm and American Diabetes Association guidelines. Height and body weight were measured using a digital scale.

### Measurement of Serum Adipocytokines and Cytokine Levels

Plasma levels of pancreatic hormones (insulin, glucagon, and C-peptide), incretins (Ghrelin, GIP, and GLP-1), adipokines (adiponectin, adipsin, resistin, leptin, visfatin, and PAI-1) and the levels of Type-1 cytokines: IFN-γ, IL-2, TNF-α, Type-17 cytokines: IL-17A, IL-22, Type-2 cytokines: IL-4, IL-5, IL-13, regulatory cytokine: IL-10 and the pro-inflammatory cytokines: IL-1α, IL-1β, IL-6, IL-12, and GM-CSF were measured using a Human Magnetic Luminex Assay kit (R&D Systems, USA) on the Bio-Rad Luminex platform (Luminex, USA), according to the manufacturer's instructions.

### Statistical Analysis

Central tendency was measured by using Geometric means (GM). Comparison between INF and UN were performed using Mann-Whitney *U*-tests with Holm's correction for multiple comparisons and the before and after treatment parameters were analyzed using Wilcoxon signed rank test. Backward stepwise methods in multiple logistic regression analysis was performed to identify factors that were influenced by *Ss* infection. Analyses were performed using Graph-Pad PRISM Version 8.0 (GraphPad, San Diego, CA) and Stata 15 (College Station, TX). JMP14 software was used to plot Principle Component Analysis (PCA).

## Results

### Study Population Characteristics

The baseline demographic characteristics and biochemical parameters are shown in Table 1. There were no significant differences in age, sex, BMI or other biochemical parameters between the two groups.

### Lower Systemic Levels of Pancreatic Hormones and Altered Levels of Incretins and Adipokines in INF Individuals

**Figure 1:**
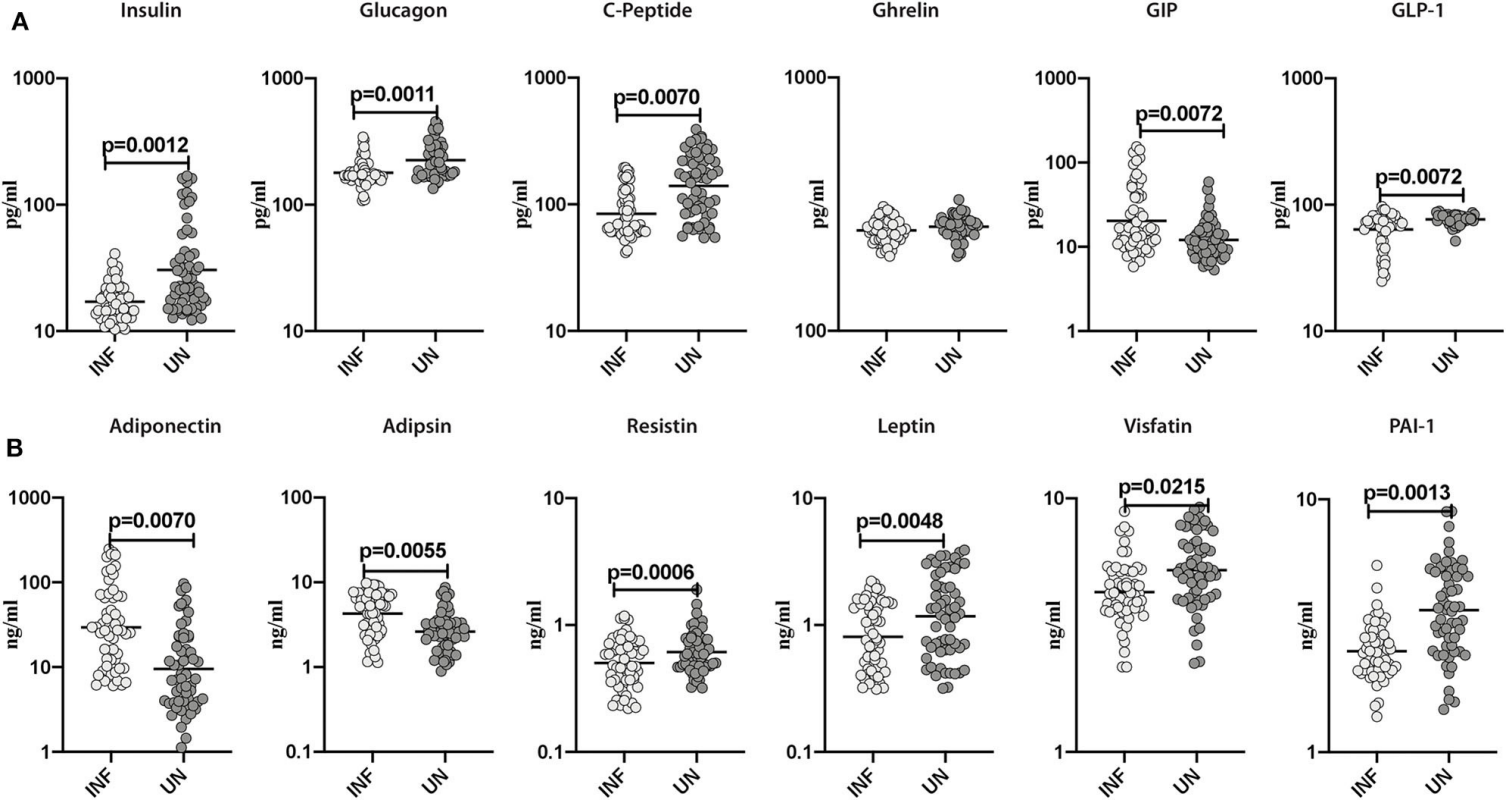
Lower systemic levels of pancreatic hormones and altered levels of incretins and adipokines in INF individuals. **(A)** Plasma levels of insulin, glucagon, C-peptide, ghrelin, GIP and GLP-1 in INF and UN individuals were measured. **(B)** Plasma levels of adiponectin, adipsin, resistin, leptin, visfatin and PAI-1 in INF and UN individuals were measured. Each dot is an individual subject with the bar representing the geometric mean (GM). Mann– Whitney *U*-test with Holms correction for multiple comparisons were done to calculate *p*-values.

To estimate the effect of *Ss* infection on pancreatic hormones (C-peptide, Insulin, and Glucagon), incretins (Ghrelin, GIP, and GLP-1) and adipokines (adiponectin, adipsin, resistin, leptin, visfatin, and PAI-1) in obesity, we assessed the levels of aforesaid parameters in INF and UN study participants. As illustrated in Figure 1A, the levels of insulin (GM of 17.09 pg/ml in INF compared to 30.45 pg/ml in UN; *p* = 0.0012), glucagon (GM of 178.7 pg/ml in INF compared to 225.4 pg/ml in UN; *p* = 0.0011), C-peptide (GM of 84.17 pg/ml in INF compared to 139.8 pg/ml in UN; *p* = 0.0070) and GLP-1 (GM of 63.69 pg/ml in INF compared to 76.71 pg/ml in UN; *p* = 0.0072) were significantly lower in INF than UN individuals. In contrast, GIP (GM of 20.39 pg/ml in INF compared to 12.07 pg/ml in UN; *p* = 0.0072) levels were significantly higher in INF compared to UN individuals. As shown in Figure 1B, resistin (GM of 0.503 ng/ml in INF compared to 0.613 ng/ml in UN; *p* = 0.0006), leptin (GM of 0.808 ng/ml in INF compared to 1.171 ng/ml in UN; *p* = 0.0048), visfatin (GM of 4.267 ng/ml in INF compared to 5.217 pg/ml in UN; *p* = 0.0215) and PAI-1 (GM of 2.51 ng/ml in INF compared to 3.66 ng/ml in UN; *p* = 0.0013) were significantly lower in INF than UN individuals. In contrast, adiponectin (GM of 29.41 ng/ml in INF compared to 9.54 ng/ml in UN; *p* = 0.0070) and adipsin (GM of 4.27 ng/ml in INF compared to 2.63 ng/ml in UN; *p* = 0.0055) were significantly higher in INF than UN individuals. Therefore, *Ss* infection is characterized by lower systemic levels of the pancreatic hormones, incretins and altered levels of adipokines in *Ss*-infected individuals with obesity.

### Lower Systemic Levels of Type-1, Type-17, and Other Pro-inflammatory Cytokines and Elevated Levels of Type-2 Cytokines in INF Individuals

**Figure 2:**
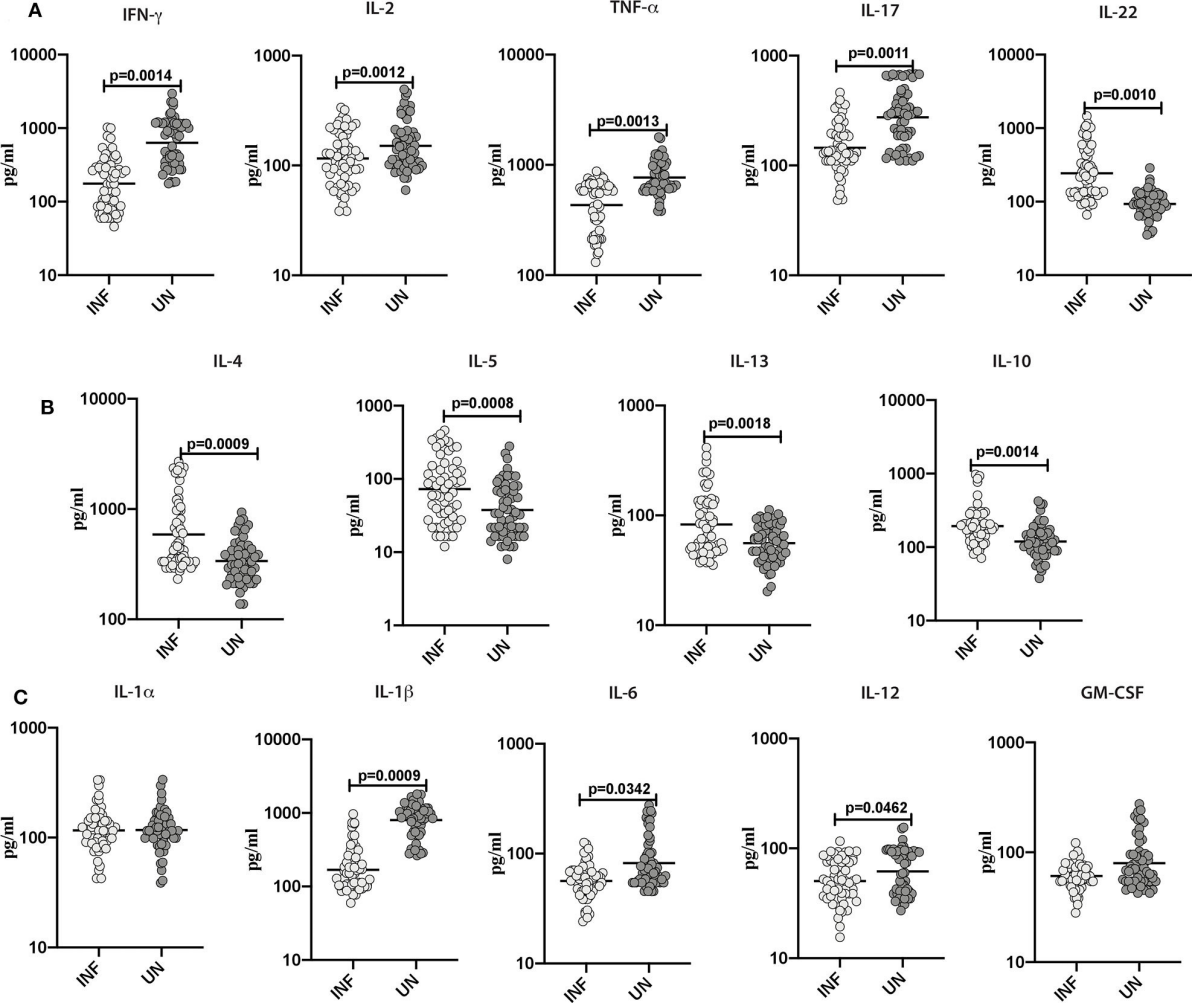
Lower systemic levels of Type-1 and Type-17 and other pro-inflammatory cytokines and higher levels of Type-2 cytokines in INF individuals. **(A)** Plasma levels of Type-1 (IFNγ, TNFα and IL-2)-, Type-17 (IL-17A and IL-22)- cytokines in INF and UN. **(B)** Plasma levels of Type-2 (IL-4, IL-5, and IL-13)- and regulatory (IL-10) cytokine in INF and UN individuals were measured. **(C)** Plasma levels of other pro-inflammatory (IL-1α, IL-1β, IL-6, IL-12, and GM-CSF) in INF and UN individuals were measured. Each dot is an individual subject with the bar representing the geometric mean (GM). Mann–Whitney *U*-test with Holms correction for multiple comparisons were done to calculate *p*-values.

Subsequently, we sought to examine the influence of Ss infection on the systemic levels of Type-1 (IFN-γ, TNF-α, and IL-2), Type-17 (IL-17A and IL-22), Type-2 (IL-4, IL-5, and IL-13), regulatory (IL-10) and pro-inflammatory cytokines (IL-1α, IL-1β, IL-6, IL-12, and GM-CSF) in INF and UN individuals. As illustrated in Figure 2A, INF individuals exhibited significantly lower levels of IFN-γ (GM of 176.2 pg/ml in INF compared to 631.6 pg/ml in UN; *p* = 0.0014), IL-2 (GM of 115.8 pg/ml compared to 150.9 pg/ml; *p* = 0.0013), TNF-α (GM of 432.2 pg/ml in INF compared to 768.8 pg/ml in UN; *p* = 0.0012), IL-17A (GM of 144.7 pg/ml in INF compared to 275.1 pg/ml in UN; *p* = 0.0087) in comparison with UN individuals. In contrast, INF individuals exhibited significantly higher levels of IL-22 (GM of 234.4 pg/ml in INF compared to 93.14 pg/ml in UN; *p* = 0.0010) when compared to UN individuals. As illustrated in Figure 2B, IL-4 (GM of 588.4 pg/ml in INF compared to 338.8 pg/ml in UN; *p* = 0.0009), IL-5 (GM of 72.71 pg/ml in INF compared to 37.79 pg/ml in UN; *p* = 0.0008), IL-13 (GM of 82.68 pg/ml in INF compared to 56 pg/ml in UN; *p* = 0.0006), and IL-10 (GM of 193.4 pg/ml in INF compared to 119.4 pg/ml in UN; *p* = 0.0007) levels were significantly lower in INF compared to UN individuals. As illustrated in Figure 2C, IL-1β (GM of 168.7 pg/ml in INF compared to 800 pg/ml in UN; *p* = 0.0009), IL-6 (GM of 56.35 pg/ml in INF compared to 82.19 pg/ml in UN; *p* = 0.0009) and IL-12 (GM of 50.39 pg/ml in INF compared to 61.89 pg/ml in UN; *p* = 0.0009) levels were significantly lower in INF than UN individuals. Thus, *Ss* infection with obesity is characterized by lower systemic levels of IL-17, Type-1 associated cytokines and pro-inflammatory cytokines and higher systemic levels of IL-22 and Type-2 associated cytokines.

### Anthelmintic Treatment Alters Plasma Levels of Pancreatic Hormones, Incretins, and Adipokines in INF Individuals

**Figure 3:**
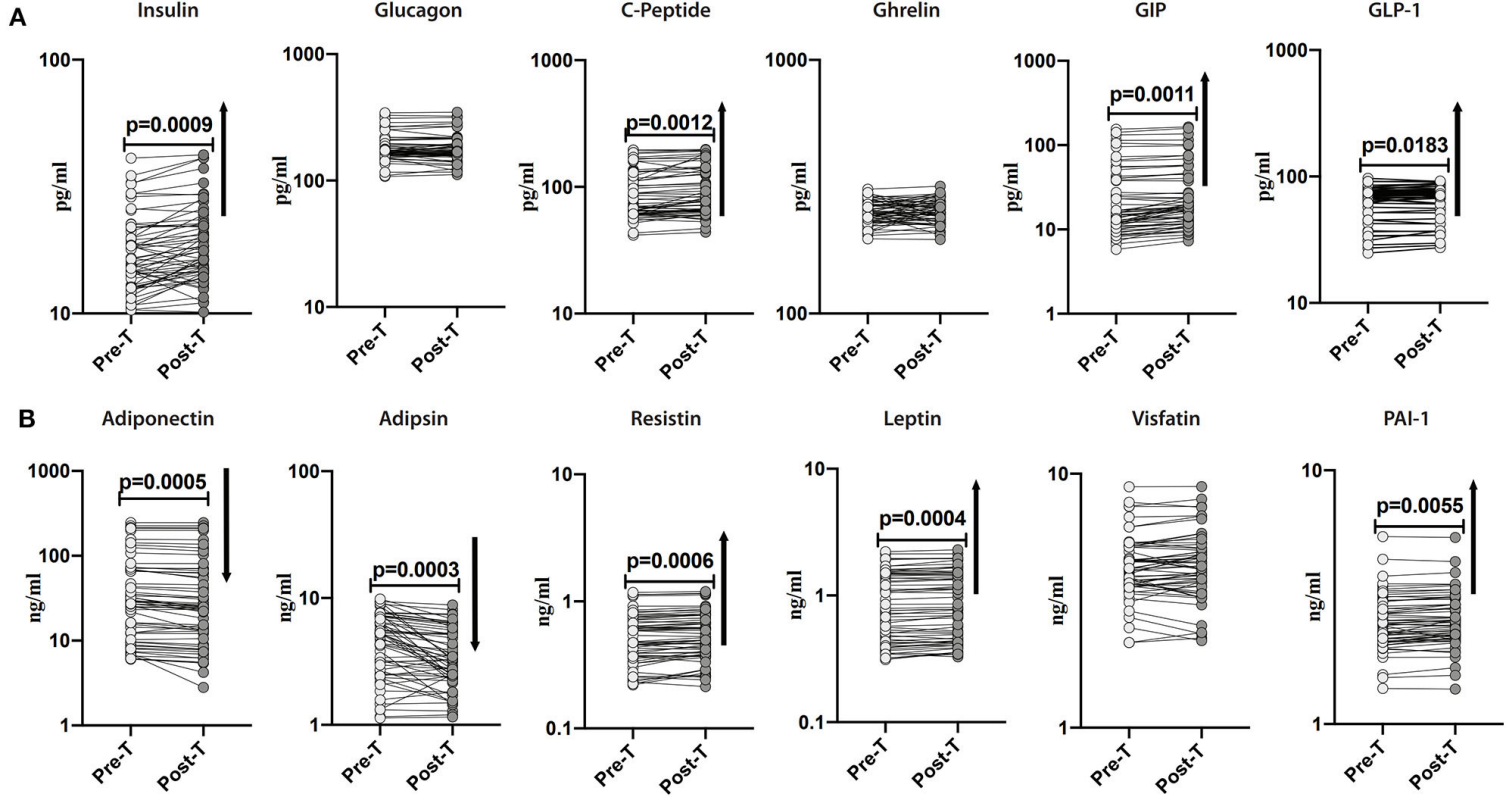
Anthelmintic treatment alters systemic levels of pancreatic hormones, incretins, and adipokines in INF individuals. **(A)** Plasma levels of insulin, glucagon, C-peptide, ghrelin, GIP, and GLP-1 in INF individuals pre-treatment [Pre-T] and 6 months following treatment [post-T] were measured. **(B)** Plasma levels of adiponectin, adipsin, resistin, leptin, visfatin, and PAI-1 in INF individuals pre-treatment [Pre-T] and 6 months following treatment [post-T] were measured. *p*-values were calculated using the Wilcoxon matched pair test.

Subsequently, we sought to examine the effect of anthelmintic therapy on the levels of pancreatic hormones, incretins and adipokines in INF individuals. For this purpose, we assessed the plasma levels of pancreatic hormones, incretins and adipokines in INF individuals following 6 months of anthelmintic therapy. As illustrated in Figure 3A, the post-treatment levels of insulin (percentage increase of 9%; *p* = 0.0009), C-peptide (percentage increase of 6%; *p* = 0.0012), GIP (percentage increase of 13%; *p* = 0.0011) and GLP-1 (percentage increase of 16%; *p* = 0.0183) were significantly increased than pre-treatment levels. As illustrated in Figure 3B, the post-treatment levels of adiponectin (percentage decrease of 11%; *p* = 0.0005) and adipsin (percentage decrease of 25%; *p* = 0.0003) were significantly decreased than the pre-treatment levels. In contrast, the post-treatment levels of resistin (percentage increase of 14%; *p* = 0.0006), leptin (percentage increase of 13%; *p* = 0.0004), and PAI-1 (percentage increase of 21%; *p* = 0.0055) were significantly increased than the pre-treatment levels. As illustrated in Supplementary Figure 1, the levels of pancreatic hormones, incretins and adipokines were not significantly different between the uninfected and post-treated individuals. Therefore, anthelmintic therapy is associated with significant alteration of pancreatic hormones, incretins and adipokines in obese individuals.

### Anthelmintic Treatment Results in Significantly Increased Levels of Type-1, Type-17 and Pro-inflammatory Cytokines and Decreased Levels of Type-2 Cytokines and IL-10 in INF Individuals

**Figure 4:**
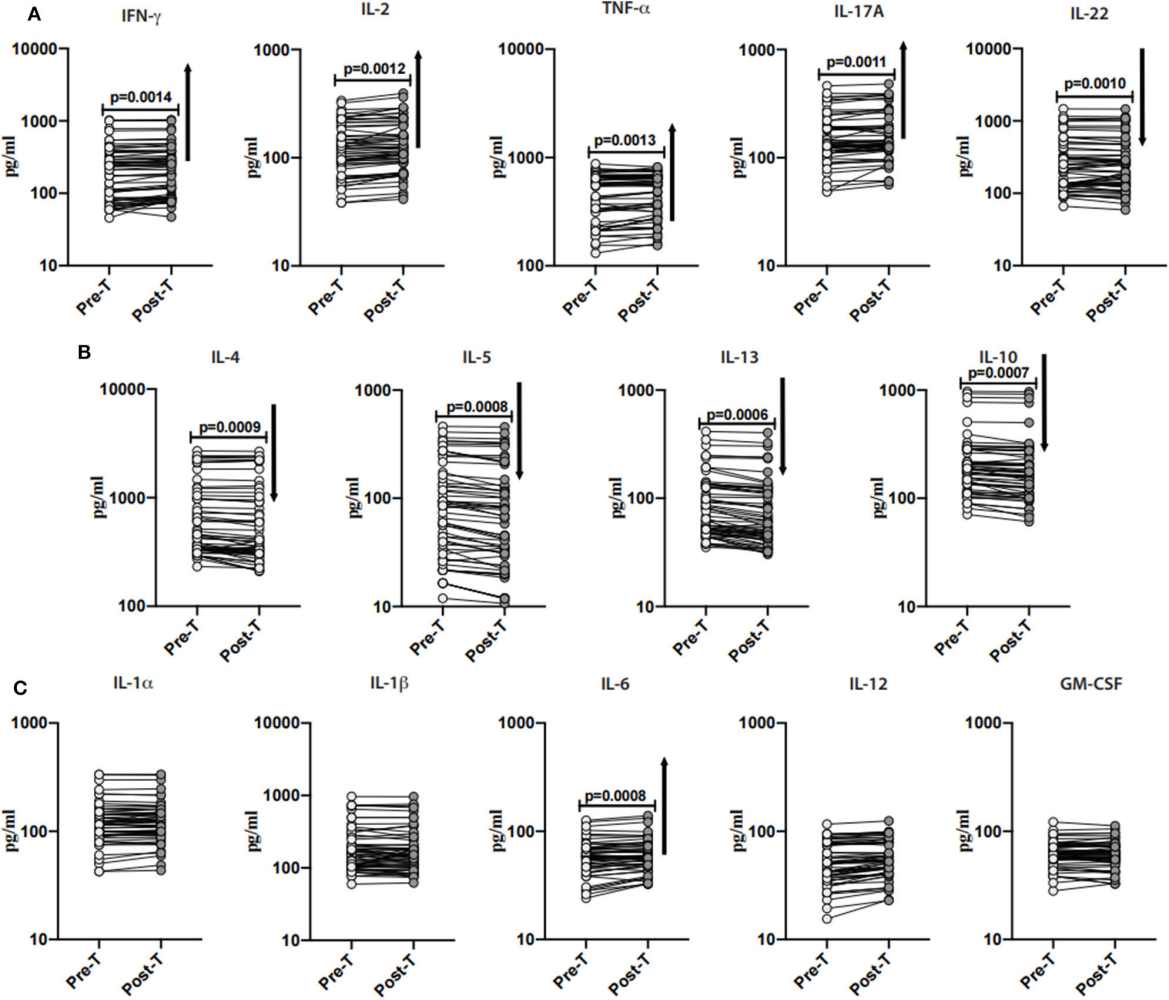
Anthelmintic treatment alters systemic levels of Type-1, Type-17, Type-2- cytokines, and IL-10 in INF individuals. **(A)** Plasma levels of Type-1 (IFNγ, TNFα, and IL-2)- and Type-17 (IL-17A and IL-22)- cytokines in INF individuals pre-treatment [Pre-T] and 6 months following treatment [post-T] were measured. **(B)** Plasma levels of Type-2 (IL-4, IL-5, and IL-13)- and regulatory (IL-10) cytokine in INF individuals pre-treatment [Pre-T] and 6 months following treatment [post-T] were measured. **(C)** Plasma levels of other pro-inflammatory (IL-1α, IL-1β, IL-6, IL-12, and GM-CSF) in INF individuals pre-treatment [Pre-T] and 6 months following treatment [post-T] were measured. *p*-values were calculated using the Wilcoxon matched pair test.

To examine the impact of anthelmintic therapy on systemic levels of Type-1 (IFN-γ, TNF-α, and IL-2)- and Type-17 (IL-17A and IL-22)- associated cytokines, we assessed the cytokines in INF individuals at baseline and following 6 months of anthelmintic therapy. As illustrated in Figure 4A, following anthelmintic treatment, the levels of IFN-γ (percentage increase of 8.3%; *p* = 0.0014), IL-2 (percentage increase of 7.9%; *p* = 0.0012), TNF-α (percentage increase of 4.6%; *p* = 0.0013), IL-17A (percentage increase of 6.4%; *p* = 0.0011) were increased, whereas IL-22 (percentage decrease of 1.2%; *p* = 0.0010) levels were decreased.

Next, to examine the impact of anthelmintic therapy on Type-2- and regulatory cytokines, we assessed the cytokines in INF individuals and baseline and following 6 months of anthelmintic therapy. As illustrated in Figure 4B, IL-4 (percentage decrease of 6.8%; *p* = 0.0009), IL-5 (percentage decease of 14.5%; *p* = 0.0008), IL-13 (percentage decrease of 12.7%; *p* = 0.0006) and IL-10 (percentage decrease of 8%; *p* = 0.0007) levels were decreased when compared to their pre-treatment levels.

Further, to examine the impact of anthelmintic treatment on pro-inflammatory cytokines, we measured the pro-inflammatory cytokines in INF individuals at 6 months following anthelmintic treatment. As shown in Figure 4C, IL-6 (percentage increase of 5.3%; *p* = 0.0008) levels were increased when compared to pre-treatment levels. Other pro-inflammatory cytokines did not show any significant difference when compared to their pre-treatment levels.

As shown in Supplementary Figure 2, the levels of Type-1, Type-17, and pro-inflammatory cytokines and Type-2 cytokines were not significantly different between the uninfected and post-treated individuals. Thus, anthelmintic treatment is associated with increased Type-1 and Type-17 associated cytokines, pro-inflammatory cytokine, IL-6, and decreased Type-2 associated cytokines in obese individuals.

### Principle Component Analysis Reveals Tendencies in Pancreatic Hormones, Incretins, Adipokines, and Cytokine Milieu in Helminth-Obese Individuals

**Figure 5:**
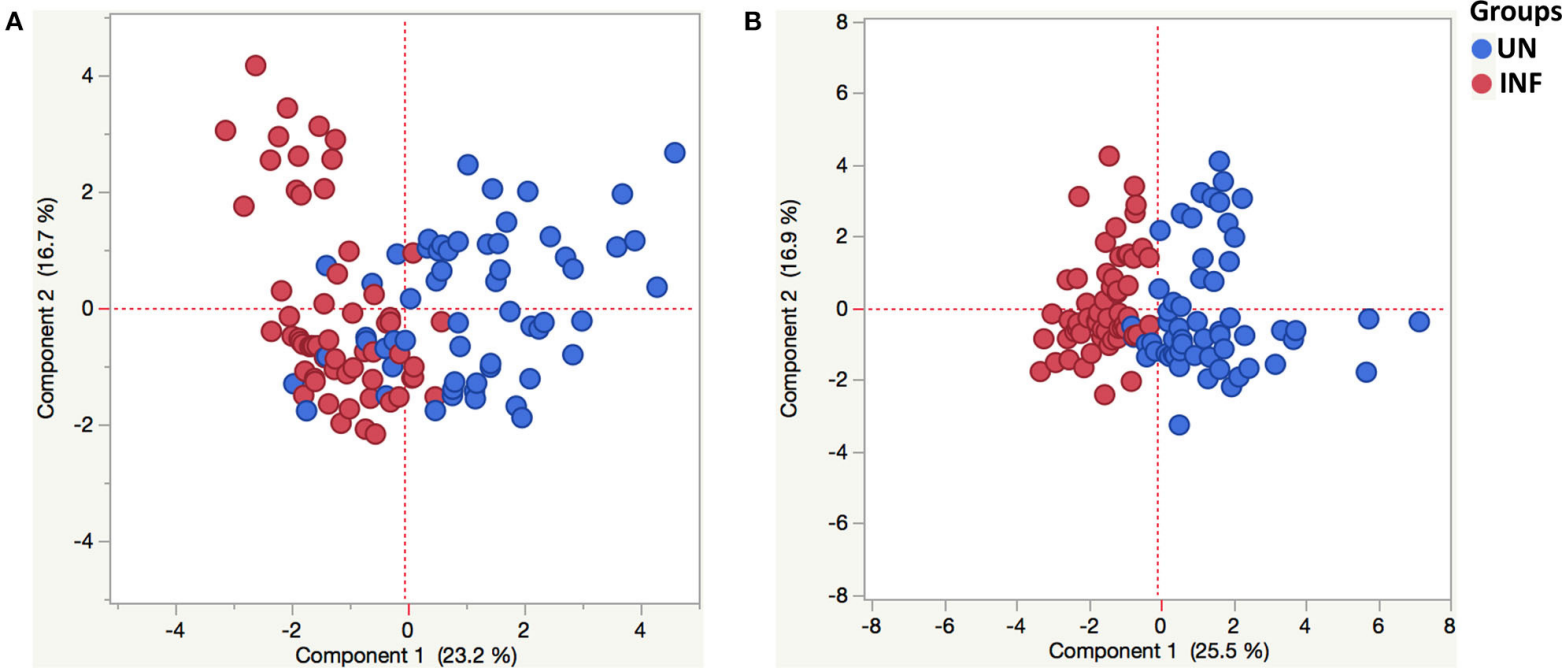
Principle component analysis reveals tendencies in pancreatic hormones, incretins, adipokines and cytokine milieu in helminth-obese individuals. Principal component analysis (PCA) was performed to show the distribution of data from the combination of two groups INF (red circles) and UN (blue circles). The PCA represents the two principal components of variation. **(A)** PCA for insulin, glucagon, C-peptide, ghrelin, GIP and GLP-1, adiponectin, adipsin, resistin, leptin, visfatin, and PAI-1in INF and UN individuals. **(B)** PCA for Type-1 (IFNγ, TNFα, and IL-2), Type-17 (IL-17A and IL-22), and other pro-inflammatory (IL-1α, IL-1β, IL-6, IL-12, and GM-CSF) cytokines of INF and UN individuals.

PCA was used to visualize differences between the groups based on the entire data set. To determine the clustering pattern of pancreatic hormones, incretins, adipokines, and cytokines between INF (red circle) and UN (blue circle) individuals, we strategized PCA with diverse inputs. As illustrated in Figure 5A, PCA analysis exhibited that pancreatic hormones, incretins, adipokines cluster differently between INF and UN individuals. The score plot of the first two components revealed 23.2 and 16.7% of overall variance, correspondingly. As shown in Figure 5B, PCA analysis of cytokines exhibited two different clusters between INF and UN individuals. The score plot of the first two components revealed 25.5 and 16.9% of overall variance, correspondingly. Thus, PCA analysis reveals the overall influence of pancreatic hormones, incretins, adipokines, and cytokine of *Ss* infection on obesity.

### Multivariate Regression Analysis of Helminth-Obesity Interaction

The influence of confounding variables on obese individuals with different analytes was evaluated in this study using multivariate regression analysis. As illustrated in Table 2, even after correcting for the influence of the age and sex, the levels of biochemical parameters such as AST, ALT and urea, RBG, HbA1c, diabetic parameters such as insulin, glucagon, C-peptide, GIP, GLP-1, adiponectin, adipsin, resistin, leptin, visfatin, and PAI-1; cytokines like IFN-γ, IL-2, TNF-α, IL-17, IL-22, IL-4, IL-5, IL-13, IL-10, IL-1β, IL-6, and IL-12 were all significantly manipulated by *Ss* infection. Therefore, our data corroborate that *Ss* infection has a great impact on numerous significant factors in obese individuals, including blood glucose levels, and the levels of the adipocytokines and the more conventional cytokines.

**Table 2:** Multiple logistic regression analysis on effect of *Ss* infection on obesity.

**Factors**	**Crude OR (95% CI)**	***p*-value**	**Adjusted OR (95% CI)**	***p*-value**
Insulin	0.268 (0.135–0.531)	<0.001	0.272 (0.138–0.537)	<0.001
Glucagon	0.114 (0.039–0.341)	<0.001	0.116 (0.039–0.345)	<0.001
C-peptide	0.279 (0.159–0.491)	<0.001	0.287 (0.164–0.500)	<0.001
Ghrelin	0.130 (0.011–1.652)	0.116	0.14 (0.011–1.822)	0.133
GIP	2.241 (1.394–3.602)	<0.001	2.213 (1.380–3.550)	<0.001
GLP-1	0.036 (0.004–0.384)	0.006	0.040 (0.004–0.402)	0.006
Adiponectin	1.827 (1.396–2.392)	<0.001	1.808 (1.389–2.354)	<0.001
Adipsin	2.545 (1.568–4.132)	<0.001	2.537 (1.566–4.111)	<0.001
Resistin	0.445 (0.235–0.843)	0.013	0.414 (0.214–0.802)	0.009
Leptin	0.615 (0.423–0.895)	0.011	0.619 (0.426–0.899)	0.012
Visfatin	0.257 (0.107–0.617)	0.002	0.254 (0.105–0.614)	0.002
PAI-1	0.123 (0.049–0.310)	<0.001	0.126 (0.051–0.316)	<0.001
IFNγ	0.275 (0.172–0.441)	<0.001	0.263 (0.162–0.429)	<0.001
IL-2	0.531 (0.322–0.877)	0.013	0.528 (0.320–0.871)	0.012
TNFα	0.092 (0.032–0.268)	<0.001	0.098 (0.034–0.282)	<0.001
IL-17	0.254 (0.145–0.447)	<0.001	0.259 (0.148–0.452)	<0.001
IL-22	12.497 (4.272–36.563)	<0.001	11.598 (3.984–33.766)	<0.001
IL-4	2.992 (1.717–5.213)	<0.001	3.108 (1.741–5.546)	<0.001
IL-5	1.640 (1.233–2.181)	<0.001	1.613 (1.212–2.145)	0.001
IL-13	2.671 (1.491–4.786)	<0.001	2.608 (1.459–4.664)	0.001
IL-10	3.202 (1.773–5.782)	<0.001	3.209 (1.770–5.819)	<0.001
IL-1α	1.005 (0.558–1.811)	0.989	1.032 (0.572–1.864)	0.918
IL1-β	0.137 (0.072–0.262)	<0.001	0.132 (0.067–0.261)	<0.001
IL-6	0.243 (0.112–0.529)	<0.001	0.239 (0.110–0.523)	<0.001
IL-12	0.481 (0.269–0.859)	0.013	0.493 (0.276–0.879)	0.016
GM-CSF	0.283 (0.130–0.616)	0.001	0.291 (0.133–0.635)	0.002

## Discussion

Helminth infections are known to limit harmful inflammatory responses and assist local and systemic metabolic homeostasis (22). Previously published reports revealed that helminths could limit the progression of metabolic diseases (23, 24), probably by altering host inflammatory responses (24). Thus, it has been proposed that a decrease in helminth infections could influence the incidence of inflammatory diseases, T2DM, obesity, insulin resistance and metabolic syndrome in many of the high income countries (25).

An earlier study showed that insulin and C-peptide levels were increased in obese individuals compared to lean controls and suggested obese subjects have an impaired glucose homeostasis and exhibit prediabetic factors, including hyperinsulinemia, and insulin resistance (26–29). Glucagon levels were elevated in obese individuals (26). Likewise, the present study has demonstrated that *Ss* infection was associated with lower systemic levels of insulin, glucagon and C-peptide levels when compared with obese without *Ss* infection and reversal following anthelmintic treatment. Recently, we have shown that *Ss* infection has a protective role on diabetes-related parameters and that *Ss* infected T2DM individuals showed decreased levels of insulin and glucagon that increased following therapy (17). Recent data also revealed that adiponectin reduces IFNγ and IL-17 responses by T cells in obese mice (30). Previously we have shown that adiponectin and adipsin levels were lowered in *Ss* infection with T2DM in comparison with *Ss* uninfected with T2DM individuals. In the present study, both adiponectin and adipsin are exhibited at elevated levels in the systemic circulation in INF individuals. These levels were significantly decreased after 6 months of anthelmintic therapy, indicating that *Ss* infection could modify the adipocytokine levels in INF individuals. Leptin induces pro-inflammatory immune responses and limits the proliferation of regulatory T cells, while adiponectin promotes the production of anti-inflammatory cytokines (31). The difference between the levels of these two adipokines has been shown to be linked with pro-inflammatory conditions and insulin resistance. A recent study revealed that leptin to adiponectin ratios in STH-infected individuals was increased following anthelmintic therapy which may in small part, lead to the moderate rise in insulin resistance (32). In our study, resistin, leptin, visfatin, PAI-1 levels were significantly lower in the INF group and increased following anthelmintic therapy. Helminths may have an impact on glucose homeostasis and insulin resistance in obesity through alternative mechanisms such as modulating the levels of adipocytokines (33, 34). This implies that adipokines have a key role in the facilitation of helminth-associated beneficial influence on insulin resistance.

Helminth infections have the ability to modulate immune responses (35). Previously, we have reported that *Ss*-infected individuals exhibited significantly decreased systemic levels of the pro-inflammatory cytokines and significantly heightened levels of the Type-2-related and regulatory cytokines (36). Type-1 related cytokines IL-1, IL-6, IL-8, IFN-γ, and MCP-1 are highly expressed in obese individuals (37). Another study showed that accumulation of inflammatory cells and adipocytes results in increased secretion of cytokines like tumor necrosis factor alpha (TNF-α) and interleukin-6 (IL-6) and lead to the progression of metabolic syndrome, consequently worsening the outcome of obesity (38). Previously, we have shown that *Ss* infection in T2DM showed significantly lower levels of Type-1 and Type-17 associated cytokines and pro-inflammatory cytokines with an increase following anthelmintic treatment (17, 18). In line with this in our study, Type-1 (IFN-γ, TNF-α, and IL-2)- and Type-17 (IL-17A)- cytokines were significantly decreased in INF individuals when compared to those without *Ss* infection. Hence, our data implies that helminth infection is characterized by the alteration of Type-1- and Type-17- cytokine responses. In our current study, IL-22 levels were increased in *Ss*-infected obese individuals and decreased following treatment. Since IL-22 is known to play a crucial role in modulating lipid metabolism and the IL-22 pathway is vital for preserving epithelial integrity, lowering chronic inflammation, and improving metabolic syndromes (39, 40), our data implies that IL-22 modulation might have a potential impact on lipid metabolism in obese individuals.

IL-4 and IL-13 are critically associated with the regulation of adipose tissue homeostasis, indicating that helminths may have an impact on metabolic status by altering adipose tissue function (41). An earlier study on *Ss* infection with T2DM showed significantly increased levels of Type-2 related cytokines (17). In our current study, an increased expression of the prototypical Type-2-associated cytokines IL-4, IL-5, IL-13, and IL-10 in *Ss*-infected obese individuals was observed. IL-10 has been shown to be able to improve inflammation in obese adipose tissue and insulin resistance induced by proinflammatory cytokines, TNF-α and IL-6 (1, 42). Various studies have determined that helminth infection and helminth- derived molecules, *S.mansoni* secreted SEA (43), *Schistosoma mansoni* egg-derived ω1 recombinant SEA (44), *L. sigmodontis* antigen (15) play an important role in metabolic disorders by stimulating a T helper 2 (Th2) response and increasing insulin sensitivity. Our data suggest that *Ss* infection could limit the inflammatory process (lower levels of pancreatic hormones, Type-I, Type-17, pro-inflammatory cytokines) by producing type-2 cytokines. This needs to be studied further mechanistically.

Taken together, our data clearly show the positive impacts of helminth infection on obesity related metabolic dysfunction. Our study has certain limitations including not determining insulin resistance directly, not having a non-obese control group and not having a placebo control group. Nevertheless, our study reveals that *Ss* infection could play a defensive role opposing the deleterious consequences of obesity by altering hormones, adipokines and the associated cytokine milieu. Certain parameters such as glucagon, visfatin and cytokines remain not significantly altered following treatment, which could perhaps reflect different kinetics in the alteration of these parameters or a lack of alteration. Our data suggest a significant association between helminth infection and the alteration of the homeostatic milieu in obesity and provide better knowledge of helminth-driven immune- and non-immune–mediated modulation of host metabolism. Our data also reinforce the possibility for helminths as a new class of biologics in alleviating inflammatory diseases and metabolic disorders. A more detailed characterization of the definitive immunomodulatory components of helminths could promote a more precise treatment approach against obesity and other inflammatory diseases.

## Data Availability Statement

All datasets generated for this study are included in the article/Supplementary Material.

## Ethics Statement

The studies involving human participants were reviewed and approved by All participants were examined as part of a natural history study protocol (12-I-073) approved by Institutional Review Boards of the National Institute of Allergy and Infectious Diseases (USA) and the National Institute for Research in Tuberculosis (India) (approval no. NCT00375583 and NCT00001230), and informed written consent was obtained from all participants. The patients/participants provided their written informed consent to participate in this study.

## Author Contributions

SB and AR conceived and planned the experiments. AR and SM executed the experiments. AR, SB, and KT analyzed the data. PM and CD provided patient samples. TN contributed reagents, materials, and analysis tools. AR, SB, and TN wrote, reviewed, and edited the manuscript. All authors contributed to manuscript revision, read, and approved the submitted version.

## Conflict of Interest

The authors declare that the research was conducted in the absence of any commercial or financial relationships that could be construed as a potential conflict of interest.

## Supplementary Material

Supplementary Figure 1 No significant differences in the systemic levels of pancreatic hormones, incretins, and adipokines between UN and Post treated individuals. **(A)** Plasma levels of insulin, glucagon, C-peptide, ghrelin, GIP and GLP-1 in UN, and Post-T individuals were measured. **(B)** Plasma levels of adiponectin, adipsin, resistin, leptin, visfatin and PAI-1 in UN and Post-T individuals were measured. Each dot is an individual subject with the bar representing the geometric mean (GM). Mann– Whitney *U*-test were done to calculate *p*-values.

Supplementary Figure 2 No significant differences in the systemic levels of Type-1 and Type-17 and other pro-inflammatory cytokines and Type-2 cytokines between UN and Post treated individuals. **(A)** Plasma levels of Type-1 (IFNγ, TNFα, and IL-2)-, Type-17 (IL-17A and IL-22)- cytokines in UN and Post-T individuals were measured. **(B)** Plasma levels of Type-2 (IL-4, IL-5, and IL-13)- and regulatory (IL-10) cytokine in UN and Post-T individuals were measured. **(C)** Plasma levels of other pro-inflammatory (IL-1α, IL-1β, IL-6, IL-12, and GM-CSF) in UN and Post-T individuals were measured. Each dot is an individual subject with the bar representing the geometric mean (GM). Mann– Whitney *U*-test were done to calculate *p*-values.
