# Supplementary figures and images for: Impact of Helminth Infection on Metabolic and Immune Homeostasis in Non-diabetic Obesity
Source: Front Immunol. 2020 Sep 16;11:2195. doi: 10.3389/fimmu.2020.02195 (PMC7524873; doi:10.3389/fimmu.2020.02195)

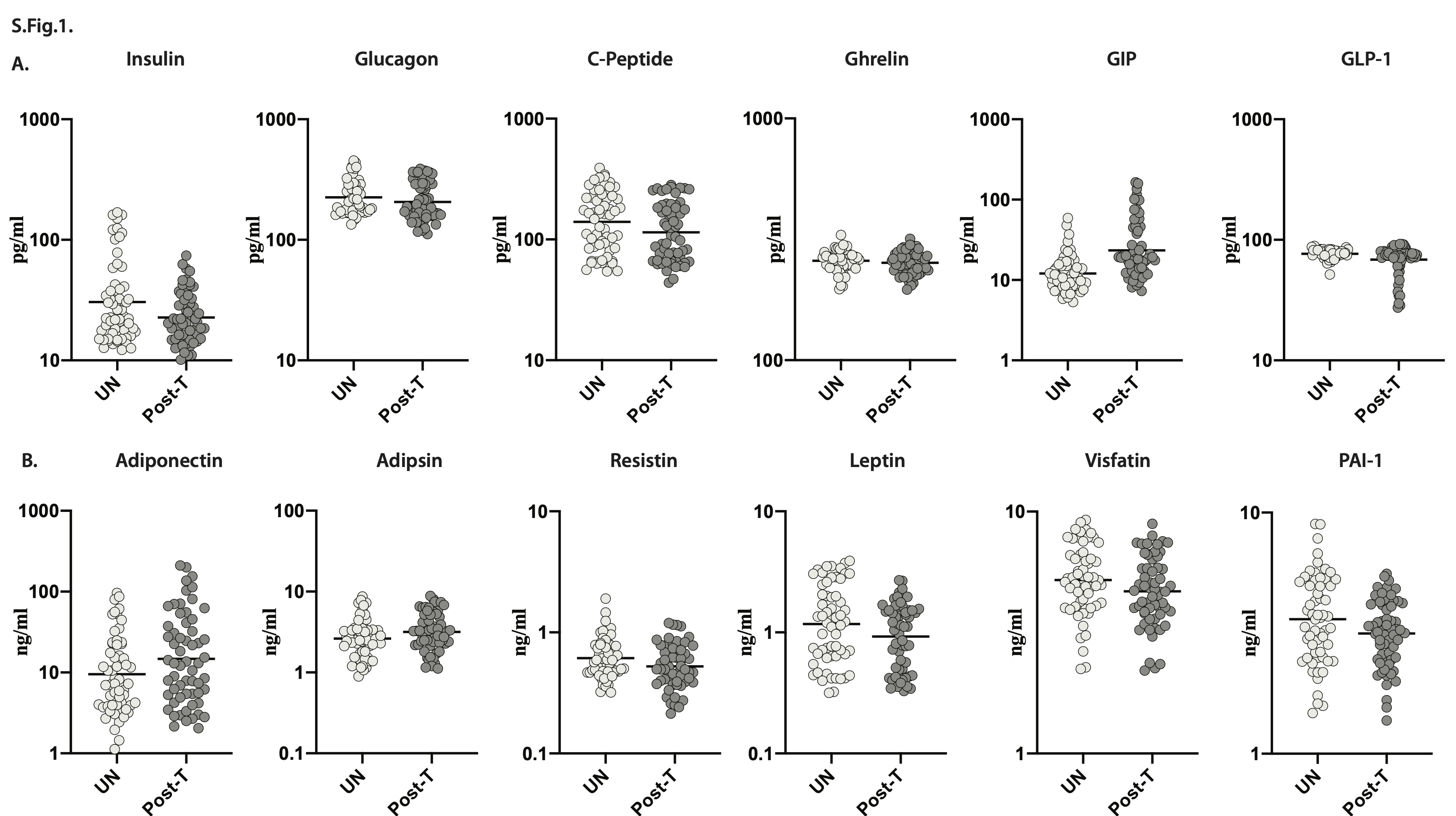

Supplement: Supplementary Figure 1 — No significant differences in the systemic levels of pancreatic hormones, incretins, and adipokines between UN and Post treated individuals. (A) Plasma levels of insulin, glucagon, C-peptide, ghrelin, GIP and GLP-1 in UN, and Post-T individuals were measured. (B) Plasma levels of adiponectin, adipsin, resistin, leptin, visfatin and PAI-1 in UN and Post-T individuals were measured. Each dot is an individual subject with the bar representing the geometric mean (GM). Mann– Whitney U-test were done to calculate p-values. [file Image_1.TIFF]

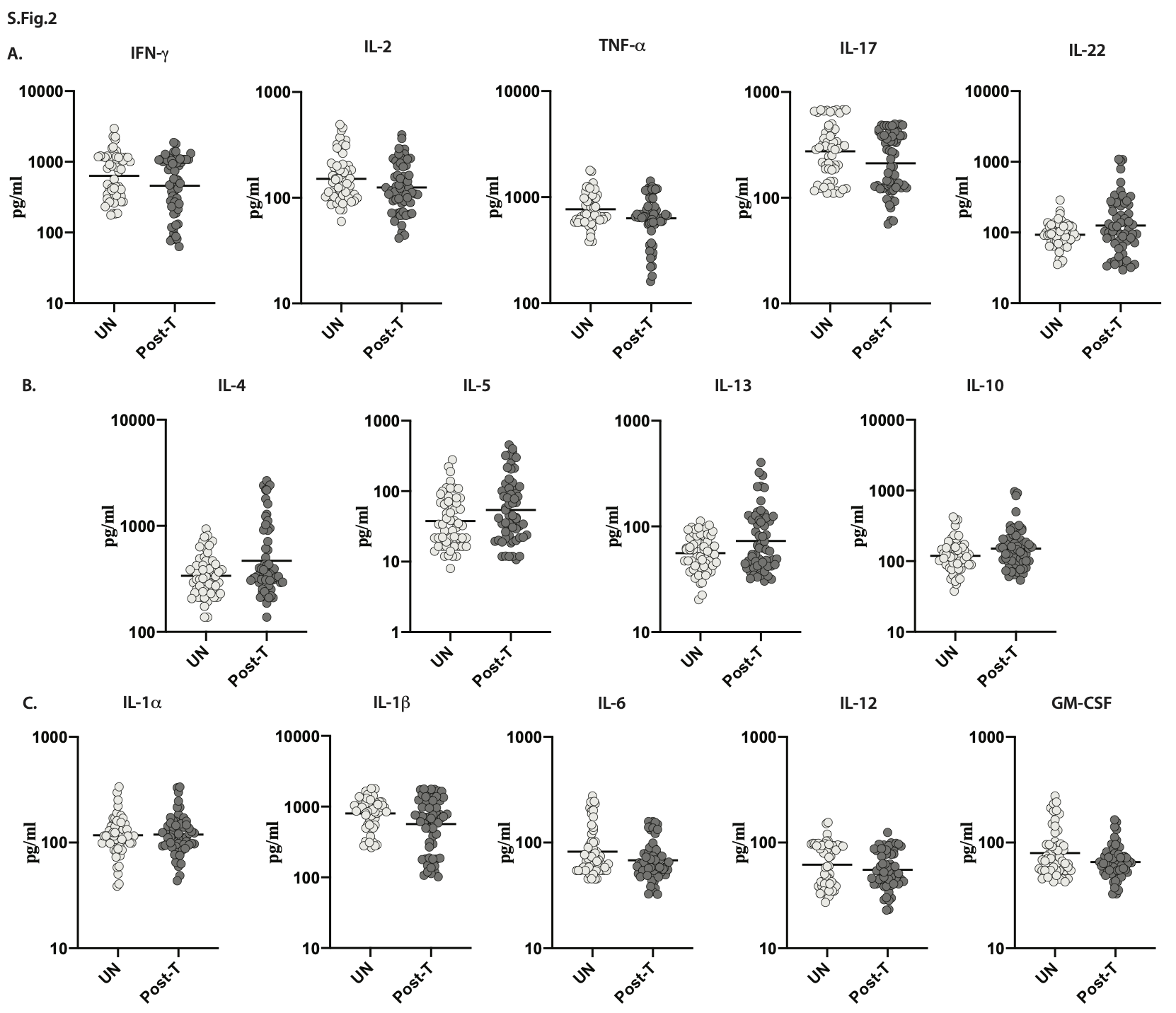

Supplement: Supplementary Figure 2 — No significant differences in the systemic levels of Type-1 and Type-17 and other pro-inflammatory cytokines and Type-2 cytokines between UN and Post treated individuals. (A) Plasma levels of Type-1 (IFNγ, TNFα, and IL-2)-, Type-17 (IL-17A and IL-22)- cytokines in UN and Post-T individuals were measured. (B) Plasma levels of Type-2 (IL-4, IL-5, and IL-13)- and regulatory (IL-10) cytokine in UN and Post-T individuals were measured. (C) Plasma levels of other pro-inflammatory (IL-1α, IL-1β, IL-6, IL-12, and GM-CSF) in UN and Post-T individuals were measured. Each dot is an individual subject with the bar representing the geometric mean (GM). Mann– Whitney U-test were done to calculate p-values. [file Image_2.TIFF]
